# Artificial Intelligence in Plastic Surgery Education: A Global Multimodel Benchmark of Large Language Models on the Plastic Surgery In-Service Training Examination

**DOI:** 10.1093/asjof/ojag052

**Published:** 2026-03-20

**Authors:** Ibrahim Güler, Gerrit Grieb, Armin Kraus, Henrik Stelling

## Abstract

**Background:**

Large language models (LLMs) are increasingly utilized in plastic surgery education. Previous studies have shown that flagship models can achieve high scores on medical examinations, including the Plastic Surgery In-Service Training Examination (PSITE). Yet evaluations often rely on single-shot accuracy of proprietary systems, neglecting stochastic variability and open-source or non-US alternatives.

**Objectives:**

The aim of this study was to comprehensively benchmark a globally representative cohort of 14 LLMs on the PSITE, assessing not only accuracy but also inter-run reliability and stochastic variability and to evaluate their role as educational tools in plastic surgery training.

**Methods:**

A cross-sectional study evaluated 7 proprietary and 7 open-source models using 100 text-based PSITE questions from the 2017-2018 examinations. Each model underwent 5 independent runs (*n* = 7000 evaluations). Performance metrics included mean accuracy (%), Fleiss’ kappa (*κ*) for reliability, and the coefficient of variation (CV) for stability. Stratified analyses assessed performance across clinical domains, proprietary vs open-source architectures, and paid vs free subscription tiers.

**Results:**

Claude Opus 4.5 (Anthropic, San Francisco, CA) (90.2%) and GPT-5.2 Pro (Open AI, San Francisco, CA) (87.0%) achieved the highest accuracy. Proprietary models significantly outperformed open-source alternatives (mean 76.1% vs 60.2%) and demonstrated superior reliability (*κ* = 0.84 vs *κ* = 0.70). Stability varied, ranging from consistent error in Falcon H1 (CV = 0.00%) to erratic instability in Mistral Medium (Mistral AI, Paris, France) (CV = 32.2%).

**Conclusions:**

Contemporary LLMs possess substantial plastic surgery knowledge, yet meaningful disparities in reliability persist. Although proprietary models currently demonstrate superior reliability as educational tools, the presence of stochastic instability necessitates cautious adoption. Accuracy alone is insufficient to judge clinical utility; stability metrics are essential for selecting AI tools in surgical education.

**Level of Evidence: 5 (Therapeutic):**

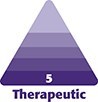

The integration of artificial intelligence (AI) into medical education is fundamentally reshaping how residents acquire and reinforce domain knowledge. Large language models (LLMs) have evolved within a few years from experimental tools into standard resources for learning, reference, and clinical brainstorming, utilized for both theoretical study and daily clinical decision support.^1-6^ Recent literature has demonstrated that flagship models can achieve passing scores on standardized medical examinations such as the United States Medical Licensing Examination (USMLE).^7-9^ Consequently, these tools have increasingly been evaluated on specialty-specific assessments, for example, the Plastic Surgery In-Service Training Examination (PSITE), which is frequently utilized as a benchmark within the field of plastic surgery. Recent studies have demonstrated that LLMs can achieve solid performance on the PSITE, often comparable to that of plastic surgery residents.^10-16^

Administered annually by the American Society of Plastic Surgeons, the PSITE comprises 250 questions covering 5 core domains: comprehensive, hand/extremities, craniomaxillofacial, cosmetic/breast, and core surgical principles.^17-19^ Beyond its role in self-assessment, the PSITE serves as a critical predictor of future success on the American Board of Plastic Surgery (ABPS) Written Examination. Previous research highlights this predictive value: residents who fail the ABPS Written Examination on their first attempt achieve a mean PSITE score of 60.1% ± 6.4%, compared with 67.0% ± 7.8% for those who pass on their first attempt.^20^ Given this strong correlation with board certification outcomes, the PSITE acts as an ideal proxy for evaluating an LLM's “domain expertise” and its potential utility as a reliable educational tool.

Existing evaluations of AI in plastic surgery remain limited in scope. Most studies have focused exclusively on proprietary flagship models (typically ChatGPT [Open AI, San Francisco, CA]) or a narrow selection of the United States centric (US-centric) systems, neglecting the rapidly expanding landscape of global AI development.^7-16^ This “monoculture” creates a blind spot for 2 factors: the distinction between proprietary and open-source ecosystems (both ranging from free and freemium to fully subscription-based services) and the issue of stochasticity. Unlike traditional deterministic databases, LLMs employ sampling-based decoding strategies that transform their deterministic architecture into a probabilistic engine, resulting in divergent outputs for identical prompts. Without assessing inter-run reliability (IRR), a high accuracy score may mask significant instability, posing risks in educational settings.^21-24^

To address these gaps, this study presents a comprehensive, cross-sectional benchmark of 14 contemporary LLMs representing a diverse global landscape, including models developed in the United States of America, China, Japan, Europe, and the United Arab Emirates. By evaluating both proprietary and open-source paradigms against the PSITE using a rigorous multirun methodology, we aim to determine not only which models possess the highest domain knowledge but also which offer the stability and reliability required for reliable integration into plastic surgery training. In this context, reliability refers to the consistency and predictability of educational output rather than patient safety. Ultimately, this study seeks to provide an evidence-based framework for selecting the most reliable AI tools in plastic surgery education.

## METHODS

### Study Design

This study employed a cross-sectional benchmarking design to evaluate accuracy (in %), IRR, and stochastic variability quantified by the coefficient of variation (CV) across contemporary LLMs on a standardized plastic surgery examination. Study design, prompt standardization, and reporting were informed by the Standards for Reporting of Diagnostic Accuracy Studies 2015 principles and by structured prompt design concepts such as the COSTAR framework (context, objective, style, tone, audience, and response), applied in accordance with the benchmark-based, nonclinical nature of the study.^25,26^ Data collection was conducted in December 2025, representing a snapshot of model performance at that specific time. All models were accessed through their official web-based interfaces, without the use of application programming interfaces or intermediary platforms, to simulate default real-world clinical use. No additional model fine-tuning, retrieval-augmented generation (RAG), or external tools were employed.

### Question Dataset

The evaluation dataset consisted of 100 manually selected multiple-choice questions drawn from the 2017 and 2018 PSITE. The use of 100 text-based PSITE questions from the 2017-2018 examination cycle was a deliberate methodological choice to ensure standardized, publicly accessible, and fully validated items. Questions requiring visual interpretation were excluded to ensure comparability across models. Questions were selected by all authors through consensus agreement. Two authors are board-certified plastic surgeons. Selection was performed to approximate a representative distribution of core plastic surgery domains (eg, facelift surgery, breast and chest wall surgery, and hand surgery). Each item presented a clinical scenario with 5 response options (A-E), of which exactly one was designated as correct according to the official PSITE answer key.

### Model Selection

**Figure 1.**
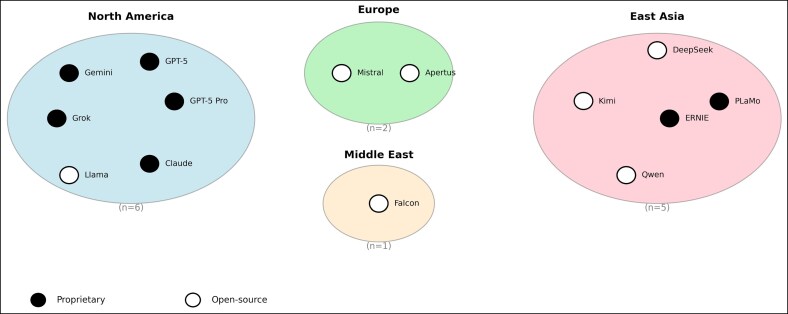
Geographic origin and licensing status of evaluated large language models: geographic distribution of the 14 evaluated large language models by region of primary development. Black markers indicate proprietary models, whereas white markers indicate open-source models. Numbers in parentheses denote the number of models per region.

Fourteen LLMs were selected to reflect the contemporary state of the art and to enable comparison between proprietary and open-source development paradigms while also providing a globally representative sample of models developed across different geographic regions and organizations (Table 1, Figure 1). Models were stratified into 2 pragmatic categories based on licensing status: proprietary (*n* = 7) and open source (*n* = 7). More granular distinctions such as fully open-source vs open-code/closed-weights or closed-code/open-weights models were not further differentiated, as these distinctions were not central to the primary performance and reliability objectives of the study.

**Table 1.** Overview of Evaluated Large Language Models.

Model	Developer	Country	License	Access
Claude Opus 4.5	Anthropic	USA	Proprietary	Paid
GPT-5.2 Pro	OpenAI	USA	Proprietary	Paid
GPT-5.2	OpenAI	USA	Proprietary	Free
Gemini 3 Pro	Google DeepMind	USA	Proprietary	Paid
Grok 4.1	xAI	USA	Proprietary	Free
ERNIE 5	Baidu	China	Proprietary	Free
PLaMo 2.1 Prime	Preferred Networks	Japan	Proprietary	Free
Llama 4	Meta	USA	Open source	Free
Kimi K2	Moonshot AI	China	Open source	Free
Falcon H1	Technology Innovation Institute	UAE	Open source	Free
Mistral Medium 3.1	Mistral AI	France	Open source	Paid
Qwen3-Max	Alibaba Cloud	China	Open source	Free
DeepSeek-V3.2	DeepSeek	China	Open source	Free
Apertus	Swiss AI Initiative	Switzerland	Open source	Free

Table summarizing the evaluated large language models, including developer, country of primary development, licensing status, and access tier at the time of evaluation.

AI, artificial intelligence; UAE, United Arab Emirates.

Model selection prioritized widely accessible, flagship or widely deployed systems with demonstrated capability for complex reasoning tasks. Notably, Mistral Medium 3.1, while proprietary, was included within the open-source comparison group to reflect its foundational origins in the Mistral open-source ecosystem and to acknowledge its role as the company's flagship reasoning-oriented model at the time of evaluation.^27,28^

### Testing Protocol

Each model was presented with the identical set of 100 examination questions across 5 independent evaluation runs. All runs were conducted using separate browser-based sessions, with no shared conversation history or retained context between runs, to ensure independence and to prevent answer carryover or memorization.

A single standardized prompt was used for all models and all runs. The prompt instructed the model to answer each question by selecting exactly 1 response option (A-E) and explicitly disallowed explanations, commentary, or follow-up questions, thereby mirroring the constraints faced by human examinees in a multiple-choice examination setting. No additional contextual information, hints, or clarifications were provided beyond the question stem itself.

### Outcome Measures

#### Accuracy

Accuracy was defined as the proportion of correctly answered questions. Model-level accuracy was reported as the mean percentage across 5 runs. Ninety-five percent CIs (95% CIs) were calculated using Wilson score intervals, based on aggregated item-level correctness across runs. This approach provides robust interval estimation for binomial outcomes without relying on normal approximation. To contextualize performance, a reference benchmark of 60% accuracy was included. This value corresponds to reported mean PSITE scores among residents who subsequently failed the ABPS Written Examination on their first attempt.^20^ This benchmark serves solely as a literature-based reference point and does not represent an official passing threshold.

#### Inter-Run Reliability (IRR)

IRR was assessed using Fleiss’ kappa (*κ*) to quantify agreement across the 5 evaluation runs, treating each run as an independent rater instance. Interpretation followed established guidelines: *κ* < 0.20 (slight), 0.21 to 0.40 (fair), 0.41 to 0.60 (moderate), 0.61 to 0.80 (substantial), and >0.80 (almost perfect agreement). Importantly, Fleiss’ *κ* measures response consistency, not correctness. A model answering incorrectly but consistently would yield a high *κ* value.^29-31^

#### Coefficient of Variation

To distinguish aggregate performance stability from item-level response consistency, the CV was calculated and expressed as a percentage.^32^ Although Fleiss’ *κ* tracks specific answer choices, CV quantifies the volatility of the model's overall accuracy across repeated sessions. This differentiation is critical, as a model could theoretically achieve a stable aggregate score despite switching answers on individual questions (low *κ*, low CV), or conversely, exhibit wide fluctuations in overall accuracy (high CV). In a clinical context, CV serves as a proxy for the predictability of model performance in a single-shot interaction.

#### Majority-Vote Accuracy

As a secondary analysis, majority-vote accuracy was calculated to simulate ensemble decision making. A strict consensus threshold was applied: an item was considered correctly answered only if the model selected the correct response in at least 3 of the 5 independent runs (≥3/5 consensus). This approach uses stochastic diversity to filter random errors, conceptually mirroring ensemble prompting but strictly maintaining identical zero-shot inputs to isolate model stability.^33^

### Statistical Analysis

Statistical analyses were designed to account for the hierarchical structure of the data (runs nested within models).

Individual model performance: For each model, mean accuracy was calculated across 5 independent runs (*n* = 500 item-level responses per model). Ninety-five percent CIs were computed using the Wilson score method. IRR was assessed using Fleiss’ *κ*, and stochastic variability was quantified using the CV. Majority-vote accuracy was defined as the percentage of items answered correctly in at least 3 of 5 runs (≥3/5 consensus).

Group-level comparisons (proprietary vs open source): Accuracy distributions were compared using run-level accuracy data (35 runs per group: 7 models × 5 runs) with the Mann–Whitney *U* test. This approach preserved statistical power derived from inter-run variability while avoiding pseudoreplication. The Mann–Whitney *U* test was selected given the non-normal distribution of accuracy scores and the ordinal nature of performance rankings.

Paired comparisons: Two prespecified pairwise comparisons were conducted: (1) Claude Opus 4.5 vs GPT-5.2 Pro (top performers; both proprietary) and (2) GPT-5.2 vs GPT-5.2 Pro (free vs paid tier). For these analyses, run-level outputs were aggregated into an item-level majority vote (correct if ≥3 of 5 runs were correct) to produce paired binary outcomes (*n* = 100 items per comparison), which were analyzed using McNemar's exact test.

Statistical significance was set at *α* = .05. All analyses were performed using Python 3.12 with standard statistical and data visualization libraries.

## RESULTS

### Overall Accuracy

**Figure 2.**
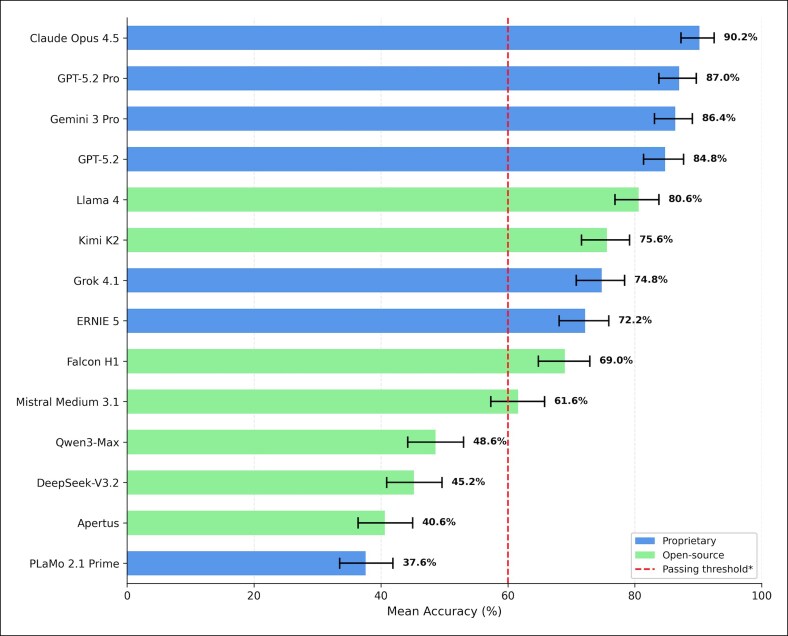
Mean accuracy with 95% CIs across evaluated large language models: mean accuracy (%) of all evaluated large language models across 5 independent runs. Error bars represent 95% Wilson score confidence intervals. (*) The dashed vertical line at 60% indicates a literature-based reference threshold corresponding to mean Plastic Surgery In-Service Training Examination performance of residents who subsequently failed the American Board of Plastic Surgery Written Examination.^20^ Models are color coded by licensing status.

Mean accuracy across the 14 evaluated models ranged from 37.6% to 90.2% (Table 2, Figure 2). Ten models (71.4%) achieved accuracy above the 60% benchmark. Performance varied substantially, with a 52.6% point spread between the highest and lowest performers.

**Table 2.** Performance Metrics for All Evaluated Models.

Model	License	Mean accuracy %	95% CI	SD	CV %	Fleiss’ *κ*	MV %
Claude Opus 4.5	Prop.	90.2	87.3-92.5	0.45	0.50	0.969	90
GPT-5.2 Pro	Prop.	87.0	83.8-89.7	2.55	2.93	0.867	90
Gemini 3 Pro	Prop.	86.4	83.1-89.1	1.52	1.76	0.873	89
GPT-5.2	Prop.	84.8	81.4-87.7	1.30	1.54	0.936	85
Llama 4	Open	80.6	76.9-83.8	1.82	2.25	0.955	82
Kimi K2	Open	75.6	71.6-79.2	1.14	1.51	0.950	76
Grok 4.1	Prop.	74.8	70.8-78.4	0.84	1.12	0.955	74
ERNIE 5	Prop.	72.2	68.1-75.9	0.84	1.16	0.795	71
Falcon H1	Open	69.0	64.8-72.9	0.00	0.00	1.000	69
Mistral Medium 3.1	Open	61.6	57.3-65.8	19.84	32.21	0.497	72
Qwen3-Max	Open	48.6	44.2-53.0	4.56	9.38	0.526	45
DeepSeek-V3.2	Open	45.2	40.9-49.6	2.59	5.73	0.299	39
Apertus	Open	40.6	36.4-45.0	0.55	1.35	0.686	40
PLaMo 2.1 Prime	Prop.	37.6	33.5-41.9	2.51	6.68	0.484	35

Performance metrics are reported as mean values across 5 independent runs per model.

CI, Wilson score confidence interval; CV, coefficient of variation; SD, standard deviation; *κ*, Fleiss’ kappa; MV, majority-vote accuracy.

Claude Opus 4.5 (Anthropic, San Francisco, CA) achieved the highest mean accuracy at 90.2%, followed by GPT-5.2 Pro at 87.0% and Gemini 3 Pro (Google, Mountain View, CA) at 86.4%. At the lower end, PLaMo 2.1 Prime (Preferred Networks, Inc., Tokyo, Japan) (proprietary) scored 37.6%, followed by Apertus (Swiss National AI Initiative) (open source) at 40.6% and DeepSeek-V3.2 (DeepSeek, Hangzhou, China) (open source) at 45.2%. Applying a majority-vote consensus (≥3/5 runs) largely confirmed the ranking order. However, discrepancies were observed in models with higher inter-run variability; notably, Mistral Medium's (Mistral AI, Paris, France) (open source) accuracy increased from a mean of 61.6% to a majority-vote score of 72%, whereas consistently low-performing models like DeepSeek-V3.2 dropped further (45.2%-39%).

### IRR and Variability

**Figure 3.**
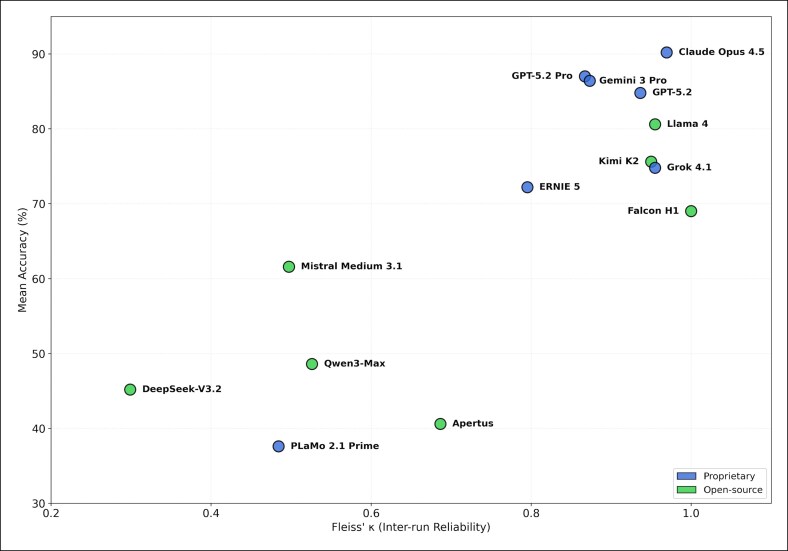
Inter-run reliability (Fleiss’ *κ*) in relation to mean accuracy: scatter plot depicting the relationship between mean accuracy (%) and inter-run reliability as measured by Fleiss’ *κ* across evaluated models. Each point represents 1 model, color coded by licensing status.

**Figure 4.**
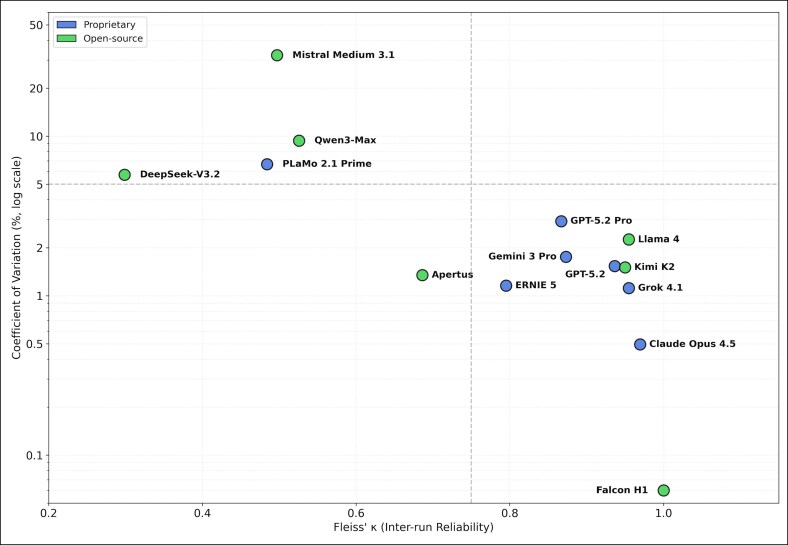
Inter-run variability (coefficient of variation) vs inter-run reliability (Fleiss’ *κ*): relationship between inter-run reliability (Fleiss’ *κ*) and stochastic variability expressed as coefficient of variation (CV). The *y*-axis is displayed on a logarithmic scale to highlight differences across stability profiles.

Substantial differences in reliability profiles were observed (Figures 3, 4). High-performing proprietary models generally demonstrated substantial to almost perfect agreement (*κ* > 0.80). In contrast, several open-source models exhibited moderate or fair agreement despite comparable mean accuracy. Falcon H1 (open source) exhibited a paradoxical profile: perfect consistency (*κ* = 1.00; CV = 0.00%) coupled with moderate accuracy (69.0%), indicating consistently reproduced errors rather than reliable correctness. Conversely, Mistral Medium 3.1 demonstrated the highest CV (32.21%), with individual run scores ranging from 39% to 79%, suggesting substantial stochastic instability.

### Item-Level Response Consistency

**Figure 5.**
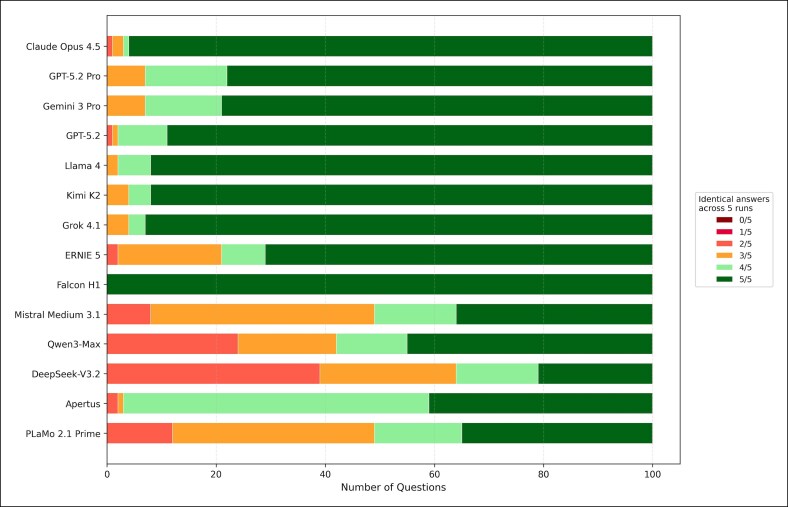
Item-level response consistency across repeated runs: distribution of item-level response agreement across 5 independent runs per model. Colors indicate the number of identical responses for each question (0/5 to 5/5), illustrating model-level stability and stochastic response behavior.

Item-level agreement patterns are summarized in Figure 5. Top-performing models (eg, Claude Opus 4.5 and GPT-5.2 Pro) demonstrated high proportions of 5/5 identical answers across runs, indicating stable knowledge retrieval. Lower-performing models (eg, Mistral Medium and PLaMo 2.1 Prime) showed frequent split decisions (eg, 2/5 or 3/5 agreement), reflecting high variability in response generation.

### License Type Comparison

**Figure 6.**
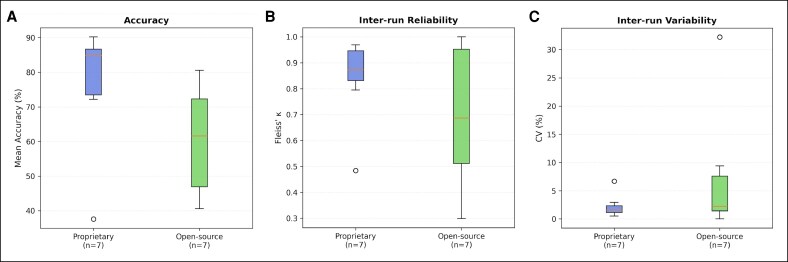
Group-level comparison of proprietary vs open-source models: (A) distribution of mean accuracy across models (Mann–Whitney *U* test, *P* < .001). (B) Inter-run reliability assessed using Fleiss’ *κ*. Differences between groups did not reach statistical significance at the model level (*P* = .65). (C) Inter-run variability expressed as coefficient of variation (CV). Group differences were not statistically significant (*P* = .46).

Proprietary models (*n* = 7) significantly outperformed open-source models (*n* = 7) in overall accuracy (Figure 6). Mean run-level accuracy was 76.1% ± 18.3% (range, 37.6%-90.2%) for proprietary models vs 60.2% ± 15.7% (range, 40.6%-80.6%) for open-source models (Mann–Whitney *U* test, *P* < .001). Median accuracy showed an even stronger disparity (84.8% vs 61.6%), highlighting the impact of lower-performing outliers on the proprietary group mean. This performance gap was observed across the distribution, with proprietary models occupying 4 of the top 6 positions.

Regarding response stability, proprietary models generally demonstrated superior consistency, although substantial within-group variability was observed. The proprietary cohort achieved a higher mean IRR (Fleiss’ *κ* = 0.840 ± 0.168; median: 0.873) compared with the open-source cohort (*κ* = 0.702 ± 0.274; median: 0.686). Similarly, stochastic variability was lower in proprietary models (mean CV = 2.2%; median: 1.5%) compared with open-source models (mean CV = 7.5%; median: 2.2%). Although these differences did not reach statistical significance (Fleiss’ *κ*: *P* = .65; CV: *P* = .46) at the model level (*n* = 7 per group).

### Pairwise Model Comparisons

To dissect performance differences between key models, 2 prespecified paired comparisons were conducted using majority-vote outcomes.

#### Top-Performer Comparison

Comparisons between the 2 highest-ranking models (Claude Opus 4.5 and GPT-5.2 Pro) revealed functional equivalence under consensus conditions. Although Claude Opus 4.5 achieved a higher mean run-level accuracy (90.2% vs 87.0%), both models attained an identical majority-vote score of 90% (90/100). Accordingly, McNemar's test demonstrated no statistically significant difference in their peak performance capability (*P* = 1.00). This suggests that the stochastic variations observed in mean scores did not translate into a tangible advantage in solved items when using an ensemble approach.

#### Subscription Tier Comparison

**Figure 7.**
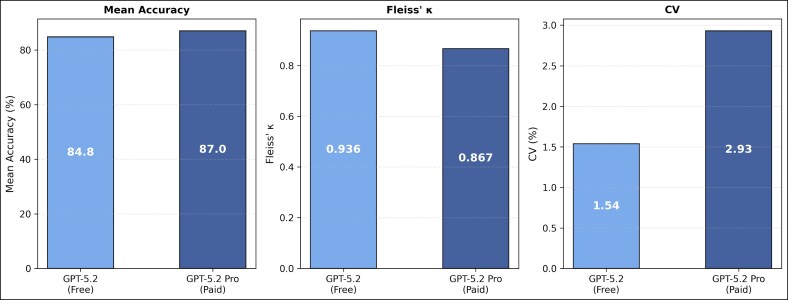
Performance comparison between free and paid subscription tiers: comparison of GPT-5.2 (free) and GPT-5.2 Pro (paid) across mean accuracy, inter-run reliability (Fleiss’ *κ*), and coefficient of variation (CV), illustrating differences between subscription tiers. Majority-vote accuracy comparison: McNemar's test, *P* = .125.

Comparison of GPT-5.2 (free) and GPT-5.2 Pro (paid) revealed subtle differences (Figure 7). Although the paid tier achieved higher mean accuracy (87.0% vs 84.8%), this difference did not reach statistical significance in the paired majority-vote analysis (McNemar's test, *P* = .125). In contrast, the free tier demonstrated superior response consistency, exhibiting higher inter IRR (Fleiss’ *κ* = 0.936 vs 0.867) and lower stochastic variability (CV = 1.54% vs 2.93%).

### Category-Specific Performance

**Figure 8.**
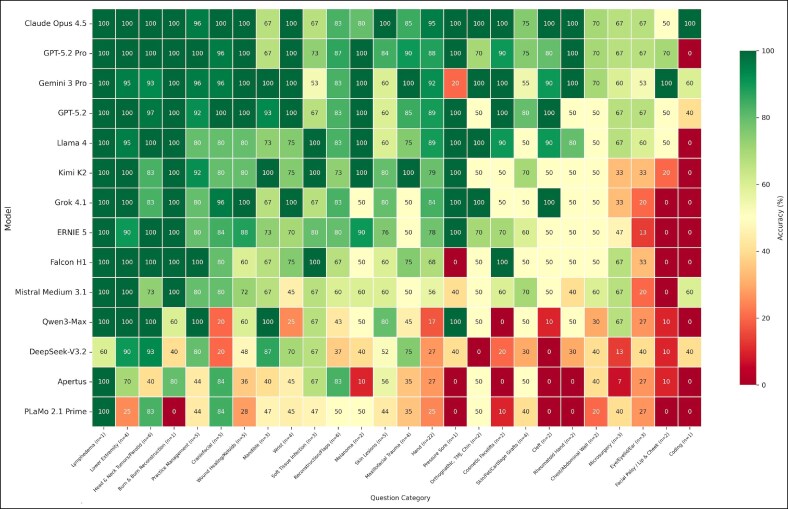
Category-specific accuracy across clinical domains: heat map illustrating category-specific accuracy (%) across clinical subspecialties for each evaluated model. Numbers within cells denote percentage of correct responses per category. Red-to-green color scale reflects increasing accuracy.

Performance analysis across clinical domains revealed distinct clustering patterns (Figure 8). High-performing models demonstrated robust competence across all categories, maintaining accuracy above 80% in most subspecialties without significant weak points. In contrast, lower-tier models exhibited erratic performance profiles, with severe deficits in complex domains such as Facial Palsy and Microsurgery. Despite high overall accuracy in certain domains, intra-model variability persisted even among top performers. Hand surgery showed strong accuracy across leading models (88%-95%), yet even among the 4 highest-performing models, split responses occurred on 9 of 22 items. High-variability questions included administrative content (eg, board certification timelines), items requiring prioritization among multiple valid clinical factors (eg, tumor recurrence risk), anatomical nomenclature, and surgical technique details where approaches vary in the literature (eg, osteotomy selection in syndromic craniosynostosis).

## DISCUSSION

This multimodel benchmark provides a comprehensive and methodologically rigorous evaluation of contemporary LLMs on the PSITE. The results demonstrate that top-tier models have reached a level of domain proficiency that rivals human trainees. Claude Opus 4.5 and GPT-5.2 Pro achieved accuracies exceeding 87%, placing them well above the historical mean PSITE score of residents passing the ABPS Written Examination on their first attempt (67.0% ± 7.8%).^20^ Under majority-vote conditions, this performance further consolidated to 90%, suggesting that ensemble strategies can effectively filter sporadic errors, particularly in models prone to stochastic instability. However, our multirun analysis reveals an important factor often overlooked in single-shot studies: although peak accuracy is high, the stability of this knowledge varies significantly between proprietary and open-source ecosystems.

### Performance Level and Educational Relevance

Across models, mean accuracy spanned a wide range, with top-tier proprietary systems achieving scores well above the reported PSITE threshold associated with subsequent board examination failure.^20^ These findings align with previous studies demonstrating that LLMs can meet or exceed passing thresholds on medical licensing and specialty examinations, including the USMLE and previous PSITE evaluations.^7-16^ However, our results extend the existing literature by demonstrating that this level of performance is not limited to a single flagship model but is observable across several contemporary architectures.

As a multiple-choice examination, the PSITE primarily assesses knowledge recall rather than clinical judgment, aesthetic reasoning, or operative decision making. Accordingly, the present findings should be interpreted as an evaluation of baseline domain knowledge and response consistency, not as a proxy for real-world clinical competence.

Educationally, and for plastic surgery residency programs in particular, these findings support the cautious integration of LLMs as supplementary learning tools rather than replacements for formal instruction. Models that consistently exceed the reference benchmark may assist residents with knowledge reinforcement, self-assessment, and question-based learning. At the same time, the observed dispersion in both accuracy and reliability shows that LLMs are not interchangeable and that indiscriminate use may expose trainees to uneven or unreliable information.^34-36^ Accordingly, this study evaluates the role of LLMs as educational tools not through pedagogical explanation but by assessing accuracy and reproducibility as necessary prerequisites for reliable knowledge reinforcement under examination-like conditions. Persistent gaps in subspecialty content and variability across systems underscore the need for cautious integration and continued human oversight. Accordingly, the integration of LLMs into plastic surgery training can be conceptualized within a human in the loop (HITL) framework, in which AI functions as a supportive but fallible educational tool requiring continuous verification by experienced surgeons.^37^

The variability of responses is not a bug, it is a feature and inherent to the operational characteristics of LLMs. Although the underlying model architectures are deterministic, real-world deployments rely on sampling-based decoding strategies that introduce stochasticity into individual responses. Combined with training on large, heterogeneous, and occasionally contradictory corpora, this mechanism explains why models may produce both variable outputs across repeated queries and stable, systematic error patterns. Consequently, average accuracy alone is insufficient to characterize educational suitability, and measures of reliability and stability are essential when considering the integration of LLMs into surgical training programs.^38-40^

Although IRR has been described in some previous evaluations of LLMs across diverse medical exam formats, the present study extends these observations by embedding variability analysis within a specialty-specific educational benchmark. By jointly assessing item-level agreement using Fleiss’ *κ* and aggregate performance stability using the CV, we are able to differentiate between models that are consistently incorrect and those that are unpredictably unstable. Importantly, our results demonstrate that the magnitude of variability differs substantially across contemporary implementations, ranging from near-perfect consistency to extreme instability, underscoring that stochastic behavior is not uniform across models and has direct implications for educational tool selection.

The observed variability across repeated runs reflects the stochastic nature of LLMs and represents a critical educational consideration. A correct response in a single instance does not guarantee stable knowledge representation, underscoring the importance of reliability metrics alongside accuracy when considering LLMs for training purposes.

### Proprietary vs Open-Source Models and Global Artificial Intelligence Diversity

A key contribution of this benchmark lies in its globally representative model selection, extending beyond the predominantly US-centric and proprietary focus of previous PSITE evaluations. Although proprietary models demonstrated superior aggregate performance and reliability, several open-source systems approached clinically meaningful accuracy thresholds, highlighting the rapid pace of development within the open ecosystem. This suggests that the extensive “reinforcement learning from human feedback” and larger parameter counts typical of closed-source commercial systems currently offer the most viable foundation for medical education.^40^ Unlike proprietary services, open-source architectures are typically not subjected to the same degree of industrial-scale alignment and safety optimization. The extensive feedback loops and guardrails inherent to commercial systems may indirectly stabilize output distributions. From an educational equity perspective, this distinction is nontrivial, as access to paid models may be limited for trainees in resource-constrained settings.^41,42^ Furthermore, because the PSITE reflects American training paradigms, model performance may partially reflect alignment with US-specific educational conventions rather than universally transferable knowledge.

However, the observed performance gap cautions against assuming functional equivalence across model classes. Until open-source systems consistently demonstrate both high accuracy and stability, their role may be best confined to exploratory learning rather than high-stakes exam preparation. Continued benchmarking will be essential as newer generations of models emerge and training paradigms evolve.

### The Reliability Paradox: Confident Hallucination vs Stochastic Instability

Beyond accuracy, our multirun analysis characterized distinct “error profiles” that have critical implications for educational reliability. We observed that accuracy metrics alone can mask dangerous behaviors. Two extremes were identified:

1. “Confident hallucination” (eg, Falcon H1): This model had a CV of 0.00% and perfect inter-run agreement (*κ* = 1.00) despite achieving suboptimal accuracy. Such models consistently reproduce the same errors across all runs. For a learner, this is particularly unreliable, as the model's consistency mimics certainty, potentially reinforcing incorrect medical concepts.^43^
2. “Stochastic instability” (eg, mistral medium): Conversely, models like mistral medium demonstrated extreme unpredictability (CV = 32.2%), with scores fluctuating between passing and failing across independent runs.

Statistically, although median stability metrics were comparable between groups, the mean stochastic variability was markedly higher in the open-source cohort (mean CV: 7.5%) compared with proprietary models (mean CV: 2.2%), driven by these unstable outliers. This confirms that generative AI acts as a probabilistic engine rather than a deterministic knowledge base.^44^ Consequently, a single “correct” answer from an LLM does not guarantee that the model possesses stable knowledge of the underlying concept, necessitating repeated measures testing for valid assessment.^45,46^

### Limitations

The results of this study must be interpreted within the context of several limitations.

1. The evaluation was restricted to text-based PSITE questions, excluding image-dependent items which are central to plastic surgery diagnosis and planning. This exclusion was a deliberate methodological choice to facilitate a comprehensive analysis of the global AI landscape. Because many contemporary models, particularly within the open-source ecosystem, currently lack multimodal capabilities, restricting the dataset to text ensured a standardized baseline comparison across all 14 architectures. Because visual reasoning constitutes a critical competency in our field, future benchmarks must incorporate multimodal capabilities, open-ended clinical scenarios, and aesthetic decision-making assessments to fully assess AI readiness.
2. The potential inclusion of older PSITE questions (2017-2018) in the models’ training datasets cannot be definitively ruled out (“data contamination”). However, the stochastic behavior observed in the majority of models, characterized by nonzero CV, argues against pure memorization and suggests a degree of generative reasoning rather than simple database retrieval. In addition, the use of older examination questions limits conclusions regarding knowledge currency. Medical knowledge and clinical recommendations evolve over time, and answers that were correct in 2017-2018 may no longer represent current best practice. Accordingly, this study does not assess whether models preferentially select outdated vs contemporaneous answers, highlighting the need for future evaluations using more recent PSITE questions.
3. All models were assessed using standardized, constrained prompts to mirror formal examination conditions. Although this zero-shot approach ensures a fair baseline comparison, it does not reflect the full potential of these systems. Alternative strategies, such as chain of thought prompting or RAG, where the model accesses external medical databases, could yield significantly different performance profiles, potentially narrowing the gap between open-source and proprietary architectures.^47,48^
4. The AI landscape is characterized by rapid and often opaque update cycles, with model capabilities shifting even within days. Consequently, this study represents a cross-sectional snapshot reflecting the performance of systems available in December 2025. Long-term durability of these rankings cannot be guaranteed as providers continuously refine model weights and safety filters.
5. Our comparison relies on historical resident performance data rather than a synchronous head-to-head evaluation. An ideal experimental framework would involve administering the PSITE to LLMs concurrently with the actual examination using never before published questions (provided no AI was used in their generation). Such a design would prevent contamination and allow for precise percentile rankings against residents stratified by postgraduate year (eg, PGY-1 vs PGY-6), offering a more granular assessment of AI expertise relative to human learning curves.
6. Although PSITE performance correlates with board examination outcomes, it remains a proxy for theoretical knowledge rather than clinical judgment or operative skill. High test scores do not equate to surgical competency. Accordingly, these findings should not be interpreted as evidence of clinical readiness, but rather as a validation of LLMs as educational tools.
7. This study focused on quantitative accuracy and reliability metrics. A qualitative analysis of the models’ generated rationales and explanatory depth, while important for educational applications, was beyond the scope of this statistical benchmark and represents a direction for future research.

## CONCLUSIONS

Contemporary LLMs demonstrate substantial baseline knowledge on the PSITE, with flagship systems achieving performance levels comparable to those associated with successful board examination outcomes. However, the significant performance gap between proprietary and open-source models, combined with the stochastic instability observed in lower-tier systems, underscores the need for caution. Meaningful differences in accuracy, reliability, and stochastic variability persist across models. Our findings confirm that accuracy alone is insufficient to characterize educational suitability and that stability metrics are essential for interpreting real-world utility. Accuracy implies competence, but stability ensures reliability. Both metrics are needed to judge AI tools in surgical education. Although top-tier proprietary models currently offer the most reliable foundation for knowledge reinforcement, the presence of stochastic variability necessitates a supervised, HITL approach to AI-assisted education.
